# Neuroimaging in Tremor

**DOI:** 10.1055/a-2942-3475

**Published:** 2026-09-16

**Authors:** Martin M. Reich, Juho Joutsa

**Affiliations:** 1 Department of NeurologyUniversity Hospital WurzburgWürzburgBavariaGermany; 2 Turku Brain and Mind Center, Clinical Neurosciences, University of TurkuTurkuSouthwest FinlandFinland; 3 Turku PET Centre, Neurocenter, Turku University HospitalTurkuSouthwest FinlandFinland

**Keywords:** tremor, computed tomography, magnetic resonance imaging, positron emission tomography, single photon emission tomography

## Abstract

Neuroimaging has been used extensively to study tremors, providing invaluable information about the underlying mechanisms and applications to clinical practice. Although isolated tremor syndromes, such as essential tremor, are generally not associated with specific neuroimaging findings, structural (MRI, CT) and molecular (PET, SPECT) brain imaging can be useful in the differential diagnostics and identifying the etiology of tremor, especially in combined tremor syndromes such as parkinsonism-associated tremors and Holmes’ tremor. In addition, brain MRI, including tractography and other connectivity measures, plays an increasing role in tremor surgery. This article will give an overview of the most frequently used clinical neuroimaging modalities, clinically relevant findings in tremor syndromes, use of neuroimaging in differential diagnostics, and use of neuroimaging in functional neurosurgery in tremors.

## Clinical Neuroimaging Modalities

Clinically used neuroimaging methods reviewed here include structural (MRI, CT) and molecular brain imaging techniques (PET, SPECT). In addition to these neuroimaging techniques, fMRI has been used extensively in research, providing valuable insight into the neurobiological mechanisms of tremors, but clinical applications of fMRI are limited. Another widely used MRI technique is tractography, which is based on diffusion-weighted imaging, but similar to fMRI, it is mainly used for research with emerging applications in presurgical planning and postsurgical deep brain stimulation (DBS) programming.

In terms of structural brain imaging, MRI provides superior tissue contrast compared to CT, making MRI the method of choice in most cases. However, MRI is associated with higher cost, more limited availability, and longer scanning times, and some patients may have contraindications for MRI, such as implanted ferromagnetic objects, electronic medical devices, or claustrophobia, in which cases CT provides a valuable alternative option. In addition, CT has a role in specific settings, such as in emergency and intensive care neurology, and preoperative investigations for MR-guided focused ultrasound (MRgFUS) ablations.

Molecular brain imaging techniques are based on injected or inhaled radiotracers, and the information provided by molecular brain imaging is dependent on the biological properties of the tracer. An enormous number of tracers have been developed, but only a small minority of them have entered into routine clinical use. In the context of tremor evaluation, they are mainly used to investigate cerebral glucose metabolism or neurotransmitter function, specifically presynaptic striatal dopamine function. The basic principles of PET and SPECT imaging are similar, but they differ in terms of the underlying physics, tracers, and hardware. PET is based on positron emission, and SPECT on single photon emitting isotope-labelled tracers. In PET, emitted positrons collide with electrons and annihilate, producing two photons emitted in opposite directions, which are then detected quasi-simultaneously by the PET detector rim. In SPECT, signal is based on emitted gamma rays detected by detectors rotating around the patient's head. Although PET provides superior spatial resolution and better opportunities for quantitative kinetic modeling compared to SPECT, it is associated with higher costs and is less widely available, which is why SPECT is currently more widely used in clinical practice.

In the diagnostic workup of tremor syndromes, and movement disorders in general, the most widely used molecular imaging techniques include glucose analog [18F]fluorodeoxyglucose ([18F]FDG) PET and dopamine transporter ligands [123I]-FP-CIT and [123I]-beta-CIT SPECT. However, there also are several other tracers that can be used as an alternative for CIT SPECT, such as tracers targeting vesicular monoamine transporter type 2 (VMAT2; e.g., [18F]DTBZ PET) and DOPA decarboxylase (DDC; e.g., [18F]fluorodopa PET), or provide complementary information in specific clinical scenarios, such as postsynaptic dopamine D2 receptor tracers [123I]-IBZM SPECT and [11C]raclopride PET. 1 In addition, molecular imaging can be useful in detecting brain tumors with high metabolic activity and tumors elsewhere using whole-body imaging such as in paraneoplastic syndromes. 2

## Clinical Neuroimaging Findings in Tremor Syndromes

In this section, we discuss the clinical use of neuroimaging techniques in the most common tremor syndromes where neuroimaging is likely to inform about the etiology of the syndrome. With some exceptions, neuroimaging is generally not necessary in typical isolated tremor syndromes unless the patient has unusual characteristics such as acute-onset and/or strictly unilateral tremor, but is more often useful in combined tremor syndromes and in cases with uncertain clinical phenomenology.

### Essential Tremor

The current diagnostic criteria of essential tremor (ET) do not incorporate any ancillary testing or biomarkers, and neuroimaging is clinically rarely used in typical cases. 3 In research settings, ET has been shown to be associated with structural and metabolic brain abnormalities, but these findings are heterogeneous and not sufficiently robust to be applicable at the individual patient level. 4 Thus, the role of neuroimaging in ET is limited to clinically uncertain cases, and structural and molecular brain imaging may be useful in cases with atypical clinical characteristics with diagnostic uncertainty to exclude some other conditions mimicking ET, such as tremors associated with neurodegenerative parkinsonism syndromes.

### Other Isolated Tremors

Neuroimaging may be helpful in identifying the etiology of isolated, segmental, postural, or kinetic, or rest tremor, as these tremors can be caused by focal brain lesions or represent early manifestations of neurodegenerative disorders. MRI is the first-line neuroimaging method, but molecular imaging can also be useful in specific settings, such as use of presynaptic dopamine function imaging using SPECT or PET in isolated rest tremor as a presenting feature of Parkinson's disease. Neuroimaging in other isolated tremor syndromes, such as task- and position-specific tremors and orthostatic tremor, is rarely beneficial. For example, orthostatic tremors manifest during standing and have not been linked to robust neuroimaging abnormalities, and neurophysiologic measures are the gold standard tool for diagnostic confirmation. Essential palatal tremor is not associated with known robust brain imaging abnormalities that could be identified using neuroimaging in individual patients. However, symptomatic palatal tremor, which is more common than essential palatal tremor, or palatal tremor as part of a combined tremor syndrome warrant brain imaging.

### Symptomatic Palatal Tremor and Myorhythmia

**Fig. 1:**
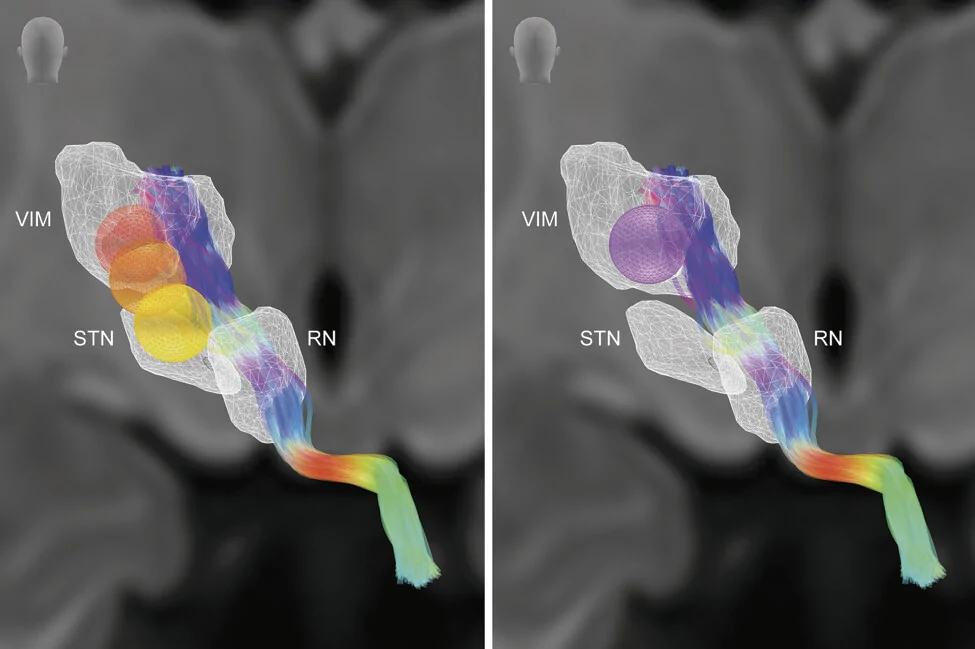
Olivary hypertrophy and hypermetabolism in progressive ataxia and palatal tremor. ( **A** ) Brain T2-weighted magnetic resonance imaging (MRI) 7 years before symptom onset showing no olivary hypertrophy. ( **B** ) Brain T2-weighted MRI 6 months after symptom onset showing olivary hypertrophy (white arrows). ( **C** ) Brain [18F]fluorodeoxyglucose positron emission tomography (FDG-PET) 3 years after symptom onset overlaid on the MRI showing olivary glucose hypermetabolism (white arrows). ( **D** ) Normal FDG-PET overlaid on the brain T2-weighted MRI (77-year-old healthy control subject). Source: Reproduce from Korpela et al 7 (CC BY).

Symptomatic palatal tremor has been reported in the context of multiple different etiologies, but is typically associated with lesions in the Guillain-Mollaret triangle and hypertrophic olivary degeneration, which can be detected using brain MRI. 5 However, it should be noted that hypertrophic olivary degeneration is not pathognomonic to symptomatic palatal tremor, as there are cases without olivary hypertrophy, and not all patients with olivary hypertrophy develop palatal tremor. In addition, other pathological processes, such as glial fibrillary acidic protein and polymerase-g mutations, and neuroferritinopathy can cause symptomatic palatal tremor and are often reflected in brain MRI, including gadolinium-enhancement and iron-sensitive sequences (SWI, T2*, QSM). Progressive ataxia and palatal tremor have also been described to be associated with olivary hypertrophic degeneration and hypermetabolism in [18F]FDG PET 6 7 ( Fig. 1 ). Similarly, myorhythmia is often associated with brain lesions involving the Guillain-Mollaret triangle.

### Holmes' Tremor

**Fig. 2:**
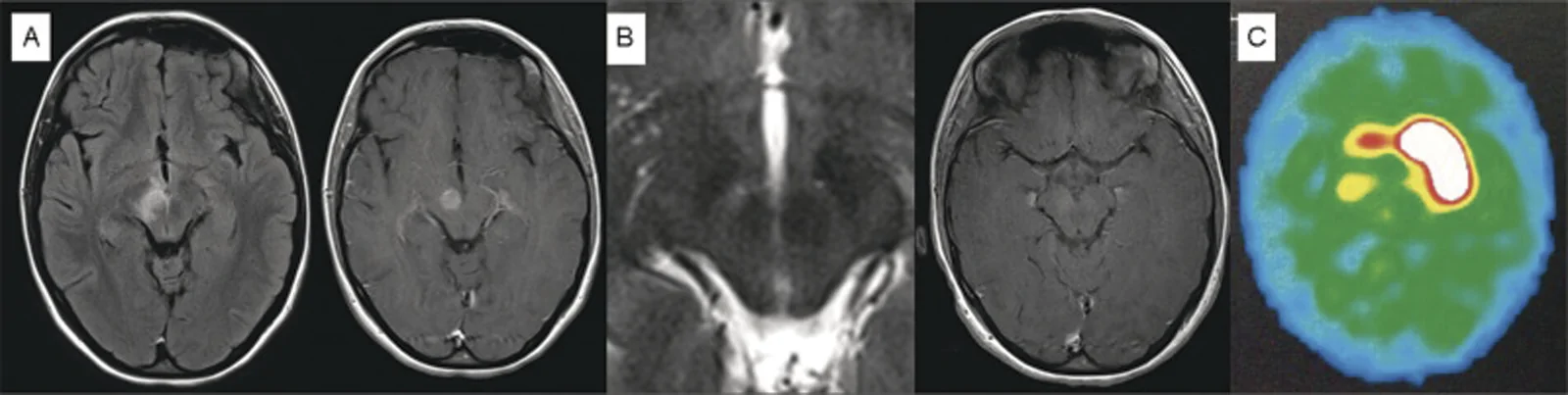
Holmes' tremor caused by an inflammatory demyelinating lesion. MRI findings. ( **A** ) Baseline: Left, transversal FLAIR image; right, transverse T1-weighted image. A 1.1 × 0.9 × 1.0-cm gadolinium-enhancing lesion in the right upper cerebral peduncle with perifocal edema is shown. ( **B** ) Follow-up MRI after 4 months: Left, only a slightly smaller right red nucleus compared to the left, and a small hyperintensity in the area of the right substantia nigra indicating discrete residual changes on T2-weighted imaging; right, complete remission on contrast-enhanced T1-weighted MRI. ( **C** ) Dopamine transporter single-photon emission computed tomography (DaT-SPECT) 5 months after symptom onset showing nearly absent DaT binding in the right striatum. Source: Reproduced from Katschnig-Winter et al (CC-BY-NC-ND). 10

Holmes' tremor is characterized by a low-frequency rest, postural and intention tremor. Holmes' tremor is rarely isolated, and it is practically always caused by brain lesions and usually associated with other neurological deficits. 8 Structural neuroimaging, preferably MRI, is necessary to identify the etiology, and thin-cut sequences through the midbrain area are recommended. Lesions causing Holmes' tremor are considered typically to involve both the nigrostriatal and dentatorubrothalamic tracts. Nigrostriatal damage is reflected in molecular imaging as a striatal presynaptic dopamine deficit using CIT-SPECT or [18F]FDOPA-PET. However, unilateral striatal dopaminergic deficit is not pathognomonic to Holmes' tremor: there are reports of Holmes' tremor with normal presynaptic function, and any lesions disrupting the nigrostriatal tract can lead to an ipsilateral presynaptic dopamine deficit 9 ( Fig. 2 ). Although more than half of Holmes' tremor patients respond to levodopa, it is not yet clear if a striatal presynaptic dopaminergic deficit predicts response to levodopa. 9 Thus, molecular imaging is not necessary in the diagnostic workup in patients with Holmes' tremor, but may be helpful in some specific cases with diagnostic uncertainty.

### Parkinsonism-Associated Tremors

**Fig. 3:**
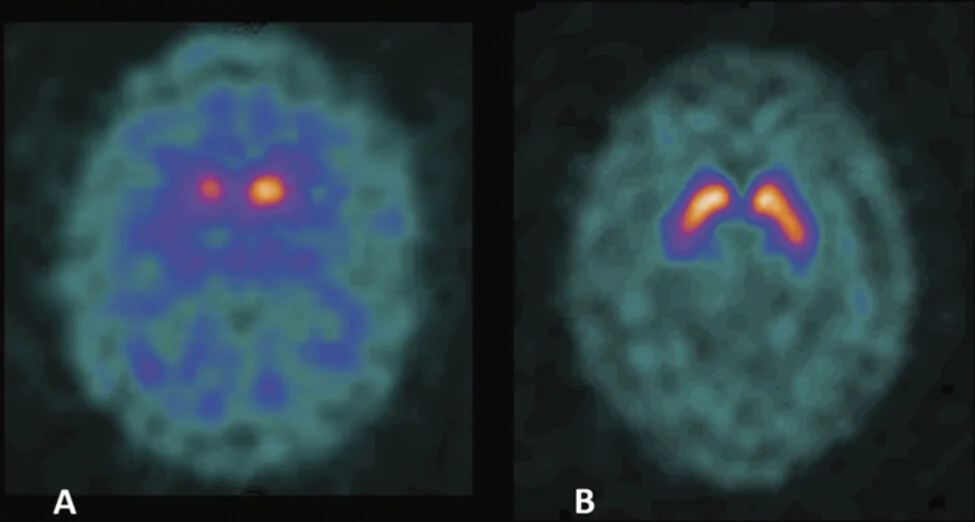
Abnormal and normal [123I]FP-CIT-SPECT scans. ( **A** ) A 52-year-old patient's DaTSCAN SPECT image showing significantly reduced, asymmetric uptake of 123I-ioflupane in the striatum. Lack of tracer accumulation bilaterally in the putamen and asymmetry of tracer uptake in the caudate nuclei with significantly lower activity on the right side. Abnormal increased tracer accumulation in the background. ( **B** ) Normal DaTSCAN SPECT reference image showing normal uptake in the striatum and background. Source: Reproduced from Balcerzak et al (CC-BY 4.0). 12

The majority of Parkinson's disease patients have rest tremor. As a presenting feature without other neurological findings, such as bradykinesia and rigidity, rest tremor can create a diagnostic challenge. In early Parkinson's disease, there are no robust structural neuroimaging abnormalities, but molecular imaging is abnormal. The most widely used imaging technique to confirm a nigrostriatal presynaptic dopamine deficit is [123I]-FP-CIT SPECT. The typical finding on SPECT is reduced tracer uptake, predominantly in the posterior putamen contralateral to the predominant motor symptoms, later progressing to a bilateral deficit ( Fig. 3 ). However, it should be noted that [123I]FP-CIT-SPECT is also abnormal in other neurodegenerative parkinsonism syndromes. [18F]FDG PET typically shows striatal hypermetabolism in Parkinson's disease, but is less sensitive and specific than presynaptic dopaminergic imaging for differential diagnostics between Parkinson's disease and ET or drug-induced parkinsonism. 11 12

**Fig. 4:**
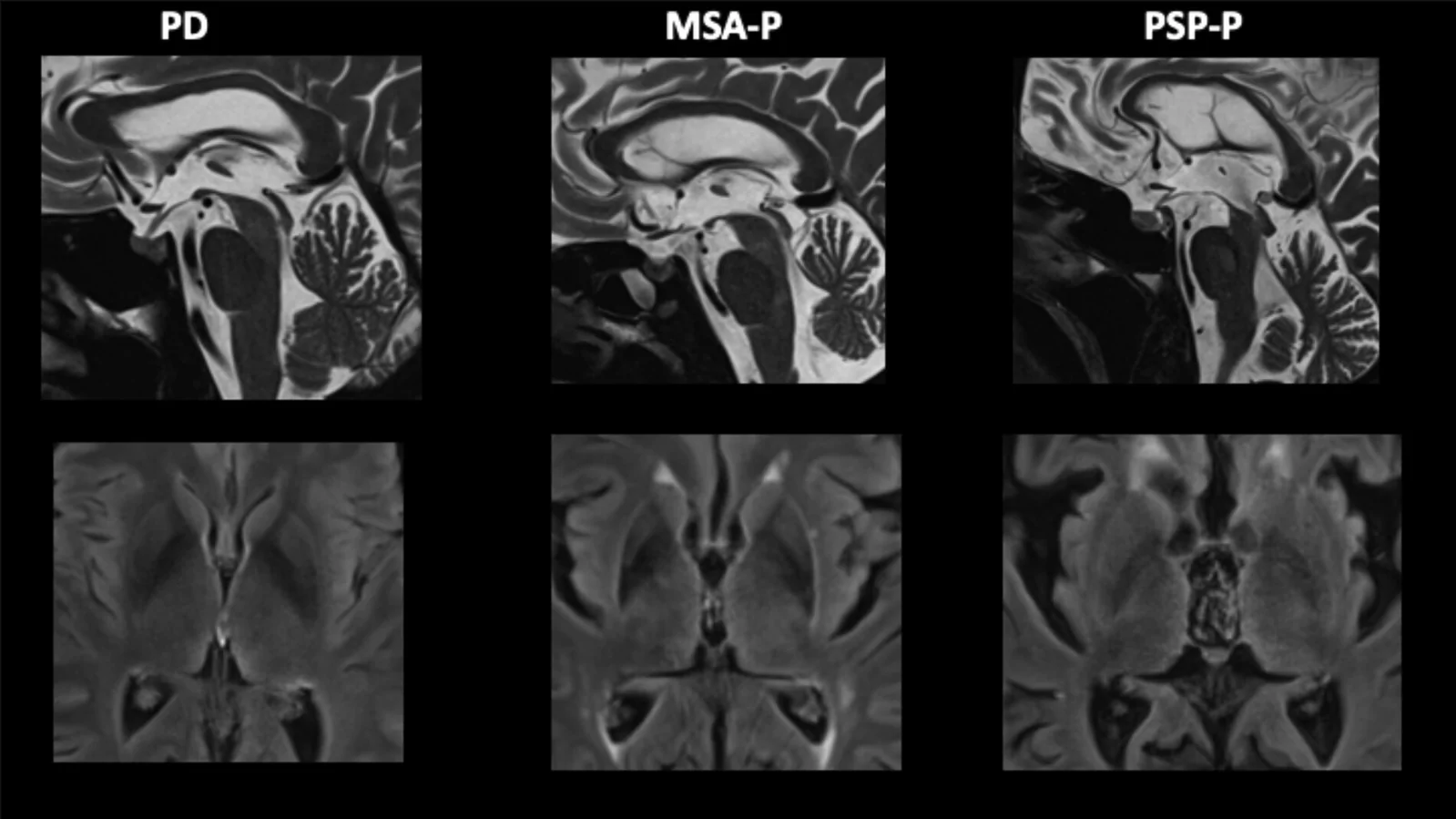
MRI features supporting the differential diagnosis of parkinsonian tremor syndromes. Conventional MRI is frequently normal in idiopathic Parkinson's disease (PD), whereas atypical parkinsonian syndromes may show characteristic structural abnormalities. In multiple system atrophy (MSA), these include putaminal atrophy and a hyperintense lateral putaminal rim in MSA-P, as well as pontocerebellar atrophy in patients with cerebellar involvement. Progressive supranuclear palsy (PSP) is characterized by midbrain atrophy, which may produce the sagittal “hummingbird” or “penguin” sign. MSA-P, multiple system atrophy parkinsonian type.

Atypical neurodegenerative parkinsonism syndromes, in which tremor is less common than in Parkinson's disease, are each associated with structural and molecular imaging abnormalities that can be helpful in the differential diagnosis. 13 For example, characteristic MRI findings in multiple system atrophy (MSA) include, for example, T2-weighted hyperintensities in the pontocerebellar tracts manifesting in the pons as the “hot cross bun sign” (typical for MSA-C) and reduced T2* or SWI signal in the globus pallidus and red nucleus with a T2 hyperintense rim around the putamen (typical for MSA-P) together with atrophy and reduced DWI white matter integrity in these structures. The most typical structural neuroimaging finding in progressive supranuclear palsy (PSP) is midbrain atrophy, manifesting as the “hummingbird sign” or “Mickey Mouse sign,” but there can also be atrophy in the pontine tegmentum and superior cerebellar peduncles ( Fig. 4 ). Although none of these findings are pathognomonic, they can provide supportive evidence or differential diagnostic clues. Unlike presynaptic dopaminergic imaging, each neurodegenerative parkinsonism syndrome has characteristic metabolic abnormalities on [18F]FDG PET, making it a useful tool in the differential diagnosis. 14

### Dystonic Tremor

Dystonias are a heterogeneous group of disorders with multiple different etiologies. Although the dystonic nature of tremor cannot be confirmed with neuroimaging methods and many dystonias have no detectable abnormalities in neuroimaging, neuroimaging can be helpful in the diagnostic workup, providing clues to the underlying etiology. 3 Generally, structural neuroimaging (MRI) is usually warranted if the etiology of dystonia is unclear. However, in typical, isolated, adult-onset cervical dystonia, routine neuroimaging is generally not considered necessary, although some experts advocate for systematic neuroimaging. 15 A recent single-center study reviewed all cases with cervical dystonias without other movement disorders with presumed idiopathic or uncertain etiology prior to brain imaging, showing that none of the 268 patients with isolated cervical dystonia had neuroimaging abnormalities that would be relevant for the etiology. 16 However, 42.9% of the 14 patients with other neurological features were found to have acquired brain lesions that were considered to be the possible or likely cause of cervical dystonia, which highlights the importance of the clinical examination and a good symptom classification before obtaining imaging.

### Combined Tremor Syndromes with Genetic Etiology

**Fig. 5:**
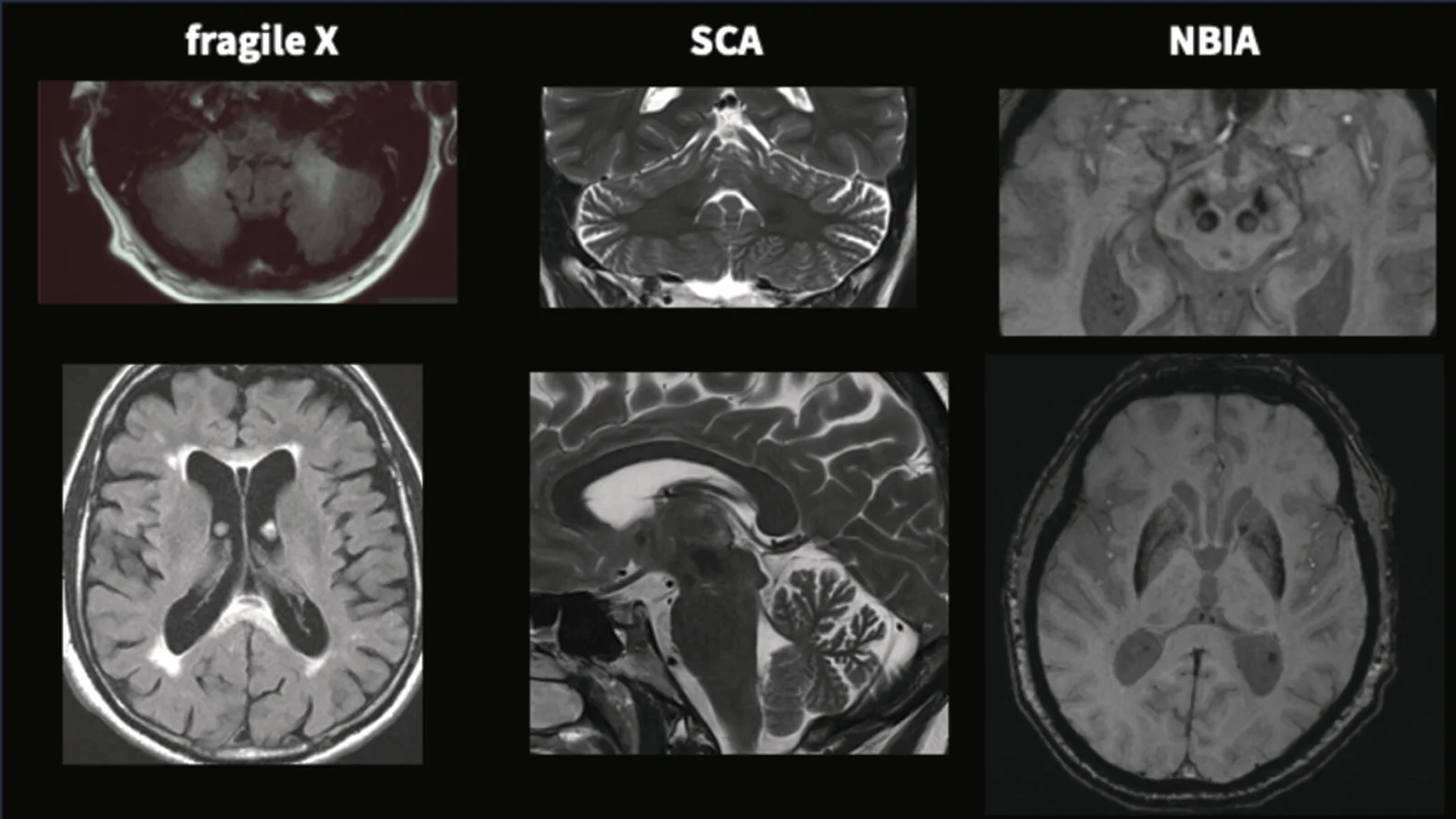
MRI findings in genetic and neurodegenerative tremor syndromes. Structural MRI can reveal characteristic abnormalities in tremor syndromes with additional neurological features. Fragile X–associated tremor/ataxia syndrome (FXTAS) is associated with cerebral and cerebellar atrophy and T2/FLAIR hyperintensities of the splenium and middle cerebellar peduncles. Spinocerebellar ataxias (SCA) show cerebellar atrophy. Neurodegeneration with brain iron accumulation (NBIA) shows abnormal iron deposition in the basal ganglia, most prominently the globus pallidus and substantia nigra, with the “eye-of-the-tiger” sign being characteristic of pantothenate kinase-associated neurodegeneration.

Neuroimaging can provide diagnostic clues for a number of genetic tremor syndromes, and these are usually not isolated tremor syndromes but combined with other neurologic or systemic symptoms and signs. However, in the early stages of the disease manifestation, tremor can be the only apparent neurological finding, creating a diagnostic challenge. The most common genetic tremor syndromes where there are characteristic neuroimaging findings include, for example, Wilson's disease, fragile X-associated tremor ataxia syndrome (FXTAS), neurodegeneration with brain iron accumulation (NBIA), and spinocerebellar ataxias (SCAs). Typical neuroimaging findings in Wilson's disease include bilateral T2 hyperintensity in the midbrain surrounding the red nucleus and substantia nigra (“face of the giant panda sign”). The characteristic findings in NBIAs include iron depositions with T2 hypointensity in the globus pallidus, creating the “eye of the tiger sign” suggestive of pantothenate kinase-associated neurodegeneration (PKAN). FXTAS is associated with T2 hyperintensity of the splenium and middle cerebellar peduncles, and SCAs with cerebellar atrophy ( Fig. 5 ). 17

## Clinical Use of Neuroimaging in the Diagnostic Workup

**Fig. 6:**
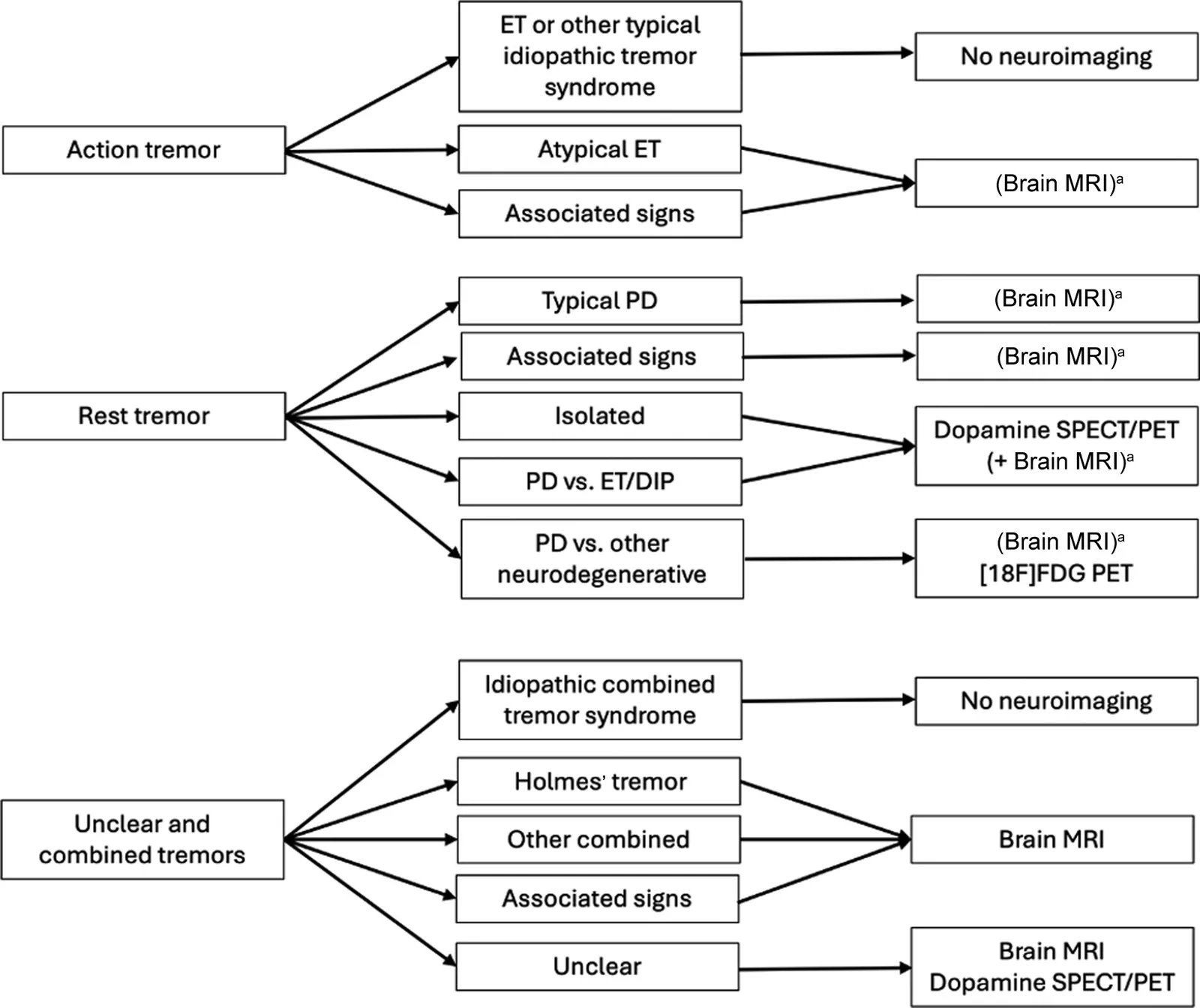
A simplified practical algorithm for use of brain imaging in common clinical scenarios. DIP, drug-induced parkinsonism; ET, essential tremor; PD, Parkinson's disease. ^a^ Brain MRI is not required for diagnosis, but may be used to exclude other etiologies.

A simplified practical algorithm representing the authors' practices for clinical use of neuroimaging is presented in Fig. 6 . This algorithm is created to address the most common clinical scenarios and is not intended as comprehensive reference for neuroimaging of tremors, as practices are variable and evidence in some scenarios is still limited.

### Structural Imaging

In typical cases of ET, the most common tremor syndrome, neuroimaging is considered unnecessary. However, if the patient has unusual characteristics, such as acute onset of symptoms, uncommon distribution of tremor or rapid progression, or is associated with other neurological symptoms and/or signs beyond those of uncertain significance in ET plus, brain MRI should usually be obtained. Similarly, neuroimaging is unlikely to be beneficial in other well-defined isolated Axis I tremor syndromes that are generally considered idiopathic, such as task- and position-specific tremors and primary orthostatic tremor. 3 This is also the case for isolated focal tremors, such as head tremor, which may develop other symptoms during follow-up and later be classified to ET or dystonic tremor in cervical dystonia. Isolated rest tremor or jaw tremor, however, can be the presenting features of Parkinson's disease, in which case dopaminergic imaging may predict development of Parkinson's disease on follow-up. 17

Contrary to isolated action tremor syndromes, combined tremor syndromes often warrant brain MRI to identify lesions or other brain abnormalities underlying the symptoms, unless the etiology can be identified with reasonable certainty. For example, the current diagnostic criteria of Parkinson's disease or adult-onset dystonias do not require brain imaging, but it is routinely performed in many centers. However, neuroimaging is acknowledged in most of the diagnostic guidelines to have potential to inform about the etiology of the symptoms. In tremor syndromes often caused by brain lesions, such as Holmes' tremor or symptomatic palatal tremor, structural brain imaging, preferably with MRI, is always warranted. Neuroimaging can also be especially helpful in cases with diagnostic uncertainty, usually starting from brain MRI and continuing to molecular imaging as needed based on the differential diagnosis.

### Molecular Imaging

**Fig. 7:**
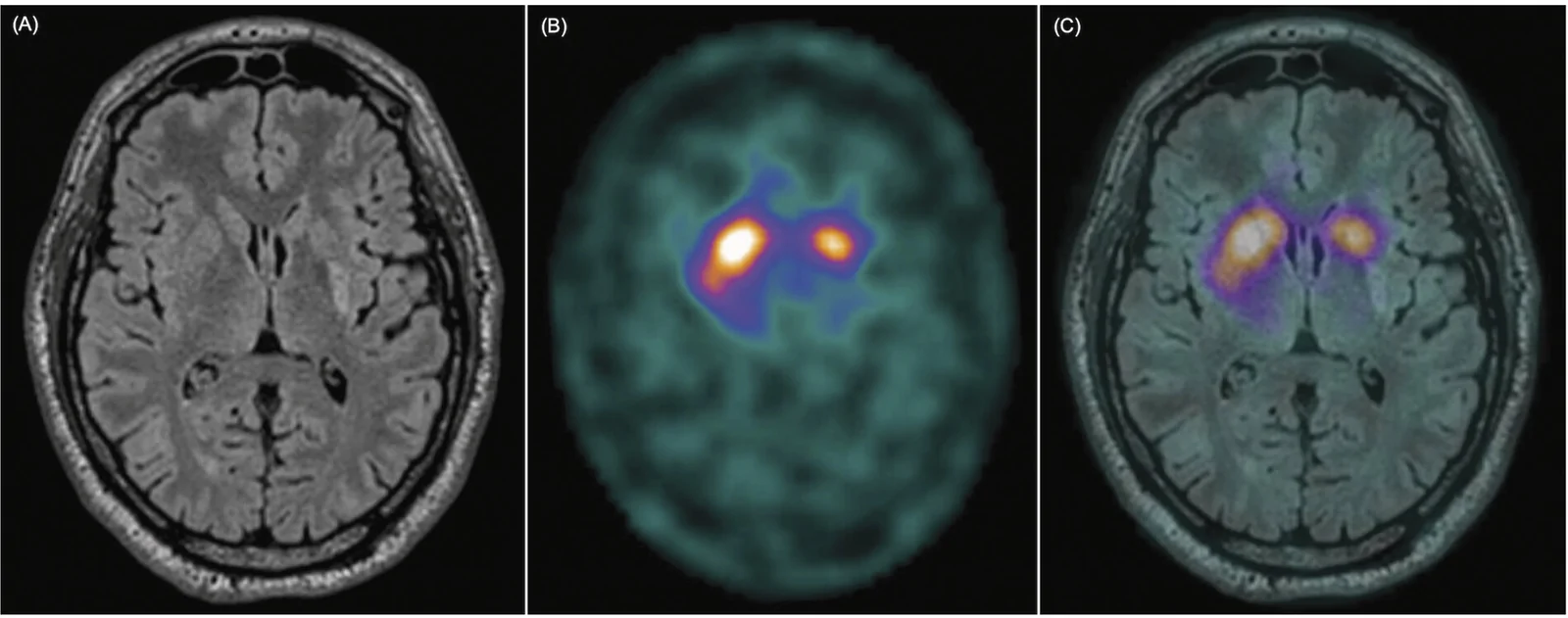
Post-stroke parkinsonism. MRI and [ ^123^ I]FP-CIT SPECT in a patient with an isolated striatal infarct. ( **A** ) T2-FLAIR-weighted image showing infarct in the left posterior putamen. ( **B** ) [ ^**123**^ I]FP-CIT SPECT showing reduced ligand uptake in the left posterior putamen. ( **C** ) Fusion of MRI and [ ^**123**^ I]FP-CIT SPECT showing that the reduced ligand uptake closely follows the limits of the vascular lesion. Source: Reproduced from Holm et al 18 (CC BY 4.0).

Prior to molecular brain imaging, structural brain imaging should usually be conducted to identify structural abnormalities that could be reflected in molecular imaging findings and influence the interpretation of the results. For example, lesions involving the nigrostriatal tract are reflected as ipsilateral striatal presynaptic dopaminergic deficits ( Fig. 7 ), which is often the case in Holmes' tremor, and any focal lesions or atrophies are also reflected as hypometabolism on [18F]FDG PET.

Presynaptic dopaminergic imaging has been validated for differentiating between Parkinson's disease and ET or drug-induced parkinsonism. There are several ligands for investigating presynaptic dopamine function, including dopamine transporter (e.g., [123I]FP-CIT, [123I]-beta-CIT, [11C]-CFT), vesicular monoamine type 2 (e.g., [11C]-DTBZ), and dopamine synthesis capacity (e.g., [18F]FDOPA). The most widely used techniques are [123I]FP-CIT-SPECT and [123I]-beta-CIT-SPECT, facilitated by relatively lower costs and better availability compared to PET imaging. All these techniques can be used to verify striatal presynaptic dopaminergic deficits already present early in the disease process, when the motor symptoms first manifest. 1 However, it should be noted that dopaminergic imaging is not required for diagnosis of Parkinson's disease, and its use should be reserved to cases with clinical diagnostic uncertainty. 19 It is also worth noting that several medications can interfere with dopamine neurotransmission, including dopamine transporter blockers, stimulants, anticholinergic drugs, antidepressants, and lithium. These medications should be discontinued at least 5 half-lives prior to imaging if possible to avoid false positives. 20

As all neurodegenerative parkinsonism syndromes can be associated with abnormal striatal dopaminergic function, dopaminergic imaging is not useful in the differential diagnosis between neurodegenerative parkinsonism. Each of these conditions, however, have their own typical patterns of metabolic abnormalities, making [18F]FDG PET suitable for differential diagnosis with reasonably high accuracy. For example, Parkinson's disease without dementia can be associated with striatal hypermetabolism, PSP with midbrain and frontal hypometabolism, MSA with hypometabolism in the striatum (particularly MSA-P) and in the thalamus, pons and cerebellum (particularly MSA-C), and cortico-basal degeneration asymmetrically in the frontal and temporal cortices. However, in ET and drug-induced parkinsonism, brain metabolism is normal. 14 21 For a review of [18F]FDG PET findings in atypical parkinsonism with illustrations, see Meyer et al. 21

## Use of Neuroimaging in Surgical Treatment

### DBS and Stereotactic Neurosurgery

Deep brain stimulation (DBS) has become the most widely used neurosurgical therapy for medically refractory tremor. Its effectiveness relies on the precise placement of electrodes within deep brain structures, traditionally the ventral intermediate nucleus (VIM) of the thalamus or the subthalamic nucleus (STN) in Parkinson's tremor, but more recently also within adjacent thalamic nuclei or connected fiber tracts. Neuroimaging is indispensable across all stages of this process. Unlike in the diagnostic workup—where imaging often plays a supportive or exclusionary role—in the surgical context, neuroimaging directly guides therapy and increasingly shapes our mechanistic understanding of how DBS exerts its effects.

Historically, stereotactic targeting relied on indirect atlas-based coordinates referenced to anatomical landmarks such as the anterior and posterior commissure. Modern imaging has transformed this approach by enabling direct visualization of some relevant nuclei (e.g., STN), reconstruction of connected fiber pathways, and integration of individualized anatomical and structural data from diffusion-based tractography into the surgical planning software. Postoperatively, imaging has become equally central. Fusion of CT and MRI datasets allows precise localization of leads, correlation of stimulation sites with clinical outcomes, and refinement of programming strategies. Functional and metabolic imaging modalities, including PET and fMRI, have extended this further by linking DBS-induced changes to distributed brain networks, providing insights into the pathophysiology of tremor and the mechanisms of its suppression.

Thus, neuroimaging in the context of DBS is not confined to anatomical targeting but represents a continuum spanning patient selection, operative guidance, postoperative validation, and mechanistic research. This integration has shifted the field from targeting isolated points to modulating distributed brain circuits, laying the foundation for increasingly personalized, network-based approaches to tremor surgery.

### Preoperative Imaging

Preoperative neuroimaging is essential to ensure both the safety and precision of DBS surgery for tremor. Modern practice goes far beyond the early atlas-based stereotactic approaches by incorporating high-resolution anatomical imaging and advanced tractography to visualize individual patient anatomy and define optimal trajectories.

Structural MRI remains the cornerstone of preoperative planning. High-field MRI allows direct visualization of deep nuclei and surrounding landmarks, including the STN, red nucleus, substantia nigra, ventricles, and vascular structures, thereby supporting both target selection and safe trajectory planning. The VIM, the classical target for tremor, cannot be sharply delineated with conventional MRI, and indirect targeting via atlas-based coordinates or AC–PC landmarks is still standard in many centers. However, structural imaging has expanded the range of potential targets beyond the VIM. In particular, the posterior subthalamic area (PSA) and zona incerta (ZI) have gained increasing attention as effective stimulation sites for tremor control. These regions contain densely packed white matter tracts—including the dentatorubrothalamic tract (DRTT)—and stimulation here often results in stronger tremor suppression compared to classical VIM targeting. 22 23 On preoperative MRI, the PSA can be indirectly localized in relation to the STN and red nucleus, although precise visualization remains challenging. 24

In Parkinson's disease, the STN has become the preferred DBS target due to its ability to address the full range of motor symptoms, including tremor. Importantly, STN stimulation is highly effective for tremor even in cases where tremor is resistant to dopaminergic medication. Structural MRI is crucial for delineating the STN and surrounding landmarks such as the substantia nigra, red nucleus, and internal capsule, as well as for planning a safe and effective trajectory. For tremor-dominant Parkinson's disease, STN targeting thus provides an alternative to thalamic or PSA/ZI approaches, with the added benefit of broader motor symptom relief. 25

Diffusion MRI and tractography have further expanded the scope of preoperative imaging by enabling *in vivo* reconstruction of white matter pathways relevant to tremor. The most prominent of these is the DRTT, which conveys cerebellar output to the thalamus and has emerged as a critical substrate in tremor generation. Tractography allows visualization of the individual DRTT trajectory and has informed a paradigm shift from purely nucleus-based to network-informed targeting. Multiple studies have shown that stimulation sites overlapping or adjacent to the DRTT are associated with superior tremor suppression, providing mechanistic support for PSA/ZI targeting and highlighting why effective tremor control can be achieved from different anatomical entry points into the same network. Despite its promise, tractography remains prone to methodological variability and requires careful interpretation. Its greatest value lies in complementing structural MRI to support both patient-specific targeting and the broader understanding of tremor circuits. 23

### Intraoperative and Immediate Postoperative Imaging

Intraoperative imaging in tremor DBS serves two central purposes: (i) ensuring accurate electrode placement and (ii) detecting potential complications such as hemorrhage. While electrophysiological mapping remains an important adjunct, imaging techniques provide the anatomical confirmation that underpins safe and effective surgery.

Fluoroscopy is the most widely used intraoperative imaging modality and remains a standard practice in nearly all centers. It allows verification of stereotactic frame position, burr hole placement, and electrode trajectory, and provides rapid confirmation of lead depth relative to planned trajectory. Although limited in resolution compared to cross-sectional imaging, its speed, accessibility, and reliability make it indispensable for routine DBS procedures.

Intraoperative CT and cone-beam CT (O-arm) have gained increasing attention in recent years. These approaches provide three-dimensional reconstructions that can be immediately fused with preoperative MRI, offering higher anatomical precision than fluoroscopy alone. Some centers have adopted O-arm imaging to reduce or even replace microelectrode recording, reporting high accuracy in lead placement. 26

Intraoperative MRI (iMRI) represents the most advanced but least commonly used option. iMRI allows real-time visualization of electrode position and surrounding anatomy, theoretically enabling direct targeting of nuclei or fiber tracts. However, due to the technical complexity, high cost, and workflow challenges, iMRI remains confined to a few specialized centers and has not been widely adopted for tremor DBS.

In most practices, intraoperative imaging is followed by an immediate postoperative CT scan, performed either in the operating room or recovery area, to confirm final lead location and exclude complications such as hemorrhage.

### Postoperative Imaging

Postoperative imaging plays a pivotal role in tremor DBS, serving both immediate clinical needs and longer-term mechanistic insights. Beyond confirming lead placement and excluding complications, postoperative imaging enables correlation of stimulation sites with clinical outcomes, mapping of symptom-specific networks, and refinement of programming strategies *.*

The primary clinical purpose of postoperative imaging is to confirm electrode placement. This is typically achieved through fusion of postoperative CT (or MRI) with preoperative MRI, providing an accurate representation of the lead trajectory and contact locations. Such reconstructions are routinely used in daily practice, particularly when programming is challenging or side effects are prominent.

**Fig. 8:**
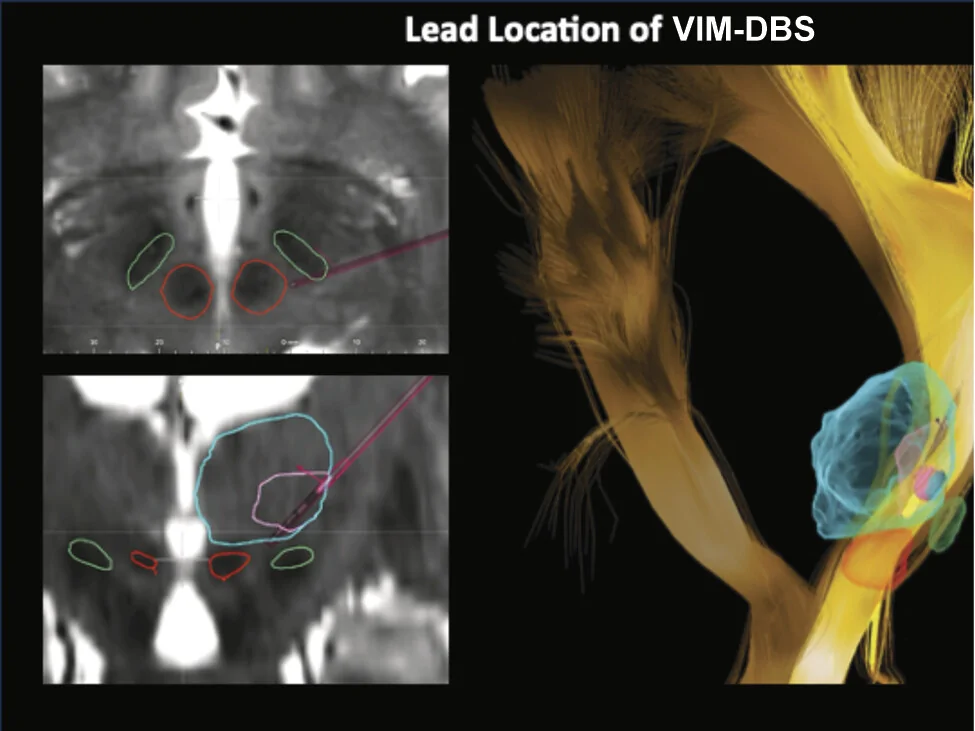
Lead localization and tract-based visualization in ventral intermediate nucleus–deep brain stimulation (VIM-DBS). Postoperative imaging allows reconstruction of DBS lead location in relation to individual anatomy and tremor-relevant fiber pathways. The left panels show lead position in axial and coronal views relative to red nucleus (red), subthalamic nucleus (STN; green), thalamus (blue), and VIM (magenta). The right panel displays a three-dimensional tractographic reconstruction, illustrating the spatial relationship between the stimulation field, nuclei, and cerebello-thalamo-cortical fibers, including the dentatorubrothalamic tract. Such imaging-based reconstructions support individualized programming by identifying contacts close to the therapeutic tremor network.

Over the last decade, commercial software platforms have brought these methods into routine clinical use. Systems such as Medtronic SureTune and Boston Scientific GUIDE XT allow visualization of individual leads in patient-specific MRI space, estimation of the volume of tissue activated (VTA), and overlay with relevant anatomical structures and white matter tracts from DTI images ( Fig. 8 ). These tools support an individualized approach: for tremor, clinicians can directly assess whether active contacts are near the atlas structures VIM, ZI, PSA, or DRTT from individual DTI, while also evaluating whether stimulation overlaps with corticospinal or medial lemniscal pathways that may cause dysarthria, gait disturbance, or paresthesias. 23 27 In this way, imaging-based programming is already impacting clinical workflows by shortening programming time, improving tremor suppression, and minimizing side effects. 28

Beyond individual anatomy, pre- and postoperative fused imaging data have been normalized into common spaces, such as MNI space, allowing probabilistic mapping of stimulation effects across larger patient cohorts. These analyses identify “beneficial spots” for tremor suppression and “avoidance areas” for stimulation-induced side effects by pooling outcomes across patients. Such probabilistic maps have yielded clinically relevant insights, showing that optimal tremor control is consistently linked to stimulation sites overlapping the DRTT or PSA, while side effects cluster near internal capsule or medial lemniscus pathways. 29 Parallel to probabilistic mapping, connectome-based analyses leverage normative structural and functional connectivity datasets to relate stimulation sites to distributed brain networks. This approach has revealed that effective tremor suppression is associated with modulation of a cerebello-thalamo-cortical network, whereas side effects are linked to connectivity with corticobulbar or limbic circuits. These findings emphasize that DBS acts by network modulation rather than isolated focal effects, 30 which is currently under validation by individual FDG-PET imaging.

While connectome analyses have thus far been primarily research tools, certain elements are beginning to move toward clinical translation. Individual DRTT targeting already represents a first pragmatic step in connectomic DBS, with tractography-based planning increasingly integrated into both commercial software and surgical workflows ( Fig. 8 ). 31 At the same time, local probabilistic mapping is the approach most likely to enter routine use in the near future. Because it builds directly on existing lead reconstruction workflows and relies on clinically validated cohort data, it has a clearer translational path than full-scale normative connectome modeling. Nevertheless, both approaches point in the same direction: a gradual shift from anatomical landmarks toward individualized, network-informed programming and imaging will play a putative role in the future of DBS treatment planning and adjustment.

### MR-Guided Focused Ultrasound

MR-guided focused ultrasound (MRgFUS) has emerged as an incision-free alternative to DBS and stereotactic neurosurgery for the treatment of medication-refractory tremor, particularly ET and tremor-dominant Parkinson's disease. Unlike DBS, which delivers chronic, modifiable electrical stimulation, MRgFUS creates a permanent lesion at the target site, most commonly the VIM of the thalamus. Recently, MRgFUS has also been approved for subthalamotomy and pallidotomy, but these targets have not yet been widely adopted for clinical use. Neuroimaging is central at every stage of the procedure, from preoperative assessment to intraoperative guidance and postoperative evaluation.

*Preoperative* imaging determines whether MRgFUS is technically feasible. A thin-slice CT of the skull is required to measure the skull density ratio (SDR), defined as the ratio of trabecular (marrow) to cortical bone density on CT. SDR reflects the efficiency of ultrasound transmission: a higher SDR indicates a more favorable skull for energy penetration, while a low SDR attenuates and scatters the ultrasound beam. Clinical trials and cohort studies have established thresholds for eligibility, typically excluding patients with SDR < 0.40 to 0.45. In a series of 189 ET patients, 28% had SDR < 0.45 and 11% had SDR < 0.40, and lower SDR was strongly associated with reduced likelihood of reaching therapeutic target temperatures intraoperatively. 32 However, more recent studies have shown that MRgFUS can usually be successfully performed even with very low SDR (<0.30) without increased risk for long-term side-effects. 33 While patients with low SDR are not absolutely excluded in all centers, outcomes are less predictable, and procedures often require higher energy delivery. MRI is also performed preoperatively to delineate target anatomy and rule out structural abnormalities that would contraindicate lesioning.

*Intraoperatively* , real-time MRI is used both for stereotactic targeting and for continuous monitoring of thermal dose delivery. This unique feature of MRgFUS enables incremental lesioning:

- Functional testing is performed with sub-therapeutic sonications, usually reaching approximately 45 to 47 °C. At these temperatures, tissue heating is reversible, allowing clinicians to test for tremor suppression and monitor for potential side effects such as dysarthria, limb ataxia, or sensory changes without creating a lesion.
- Therapeutic lesioning is then achieved with higher-energy sonications that elevate the focal temperature to 50 to 54 °C, corresponding to an accumulated thermal dose of approximately 100 to 200 CEM43 (equivalent minutes at 43 °C). This range reliably produces a permanent focal lesion, while minimizing risk to surrounding structures.

Thermal mapping with MRI ensures safety by verifying that heating remains confined to the intended target and detecting any spread toward adjacent tissue, providing both precision and real-time safety control.

**Fig. 9:**
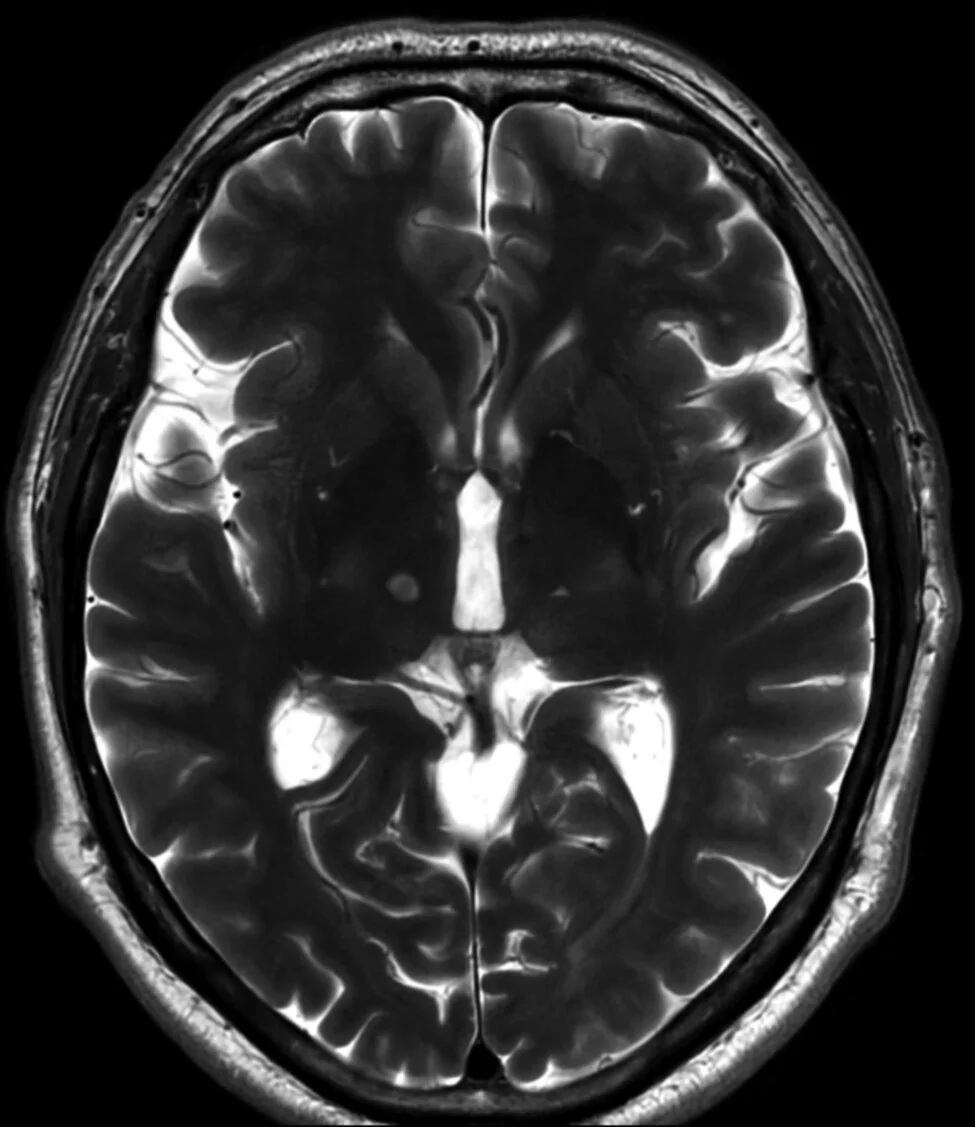
Bilateral thalamotomy lesions using MR-guided focused ultrasound (MRgFUS). MRgFUS thalamotomy lesions a month (left side) and a year (right side) after the procedure.

*Postoperative MRI* provides direct visualization of the lesion. Acutely, the lesion appears as a sharply circumscribed focus of restricted diffusion and T2 hyperintensity. Within days to weeks, this evolves into a well-demarcated cavitated lesion surrounded by a rim of gliosis. Typical lesion diameters range from 4 to 6 mm on follow-up MRI ( Fig. 9 ), though lesion size and shape vary with the cumulative thermal dose and target location. Importantly, both tremor improvement and adverse effects correlate strongly with lesion topology: lesions placed slightly more ventral or medial often increase the risk of dysarthria or imbalance. Recent work has demonstrated that variability in lesion placement within the thalamus explains much of the heterogeneity in clinical outcomes, reinforcing the importance of precise image-guided targeting. 34 It should also be noted that the size of thalamotomy lesion on MRI is not stable, as it often shrinks with time ( Fig. 9 ). However, this seems to be a radiological phenomenon and does not correlate with loss of therapeutic benefit.

**Fig. 10:**
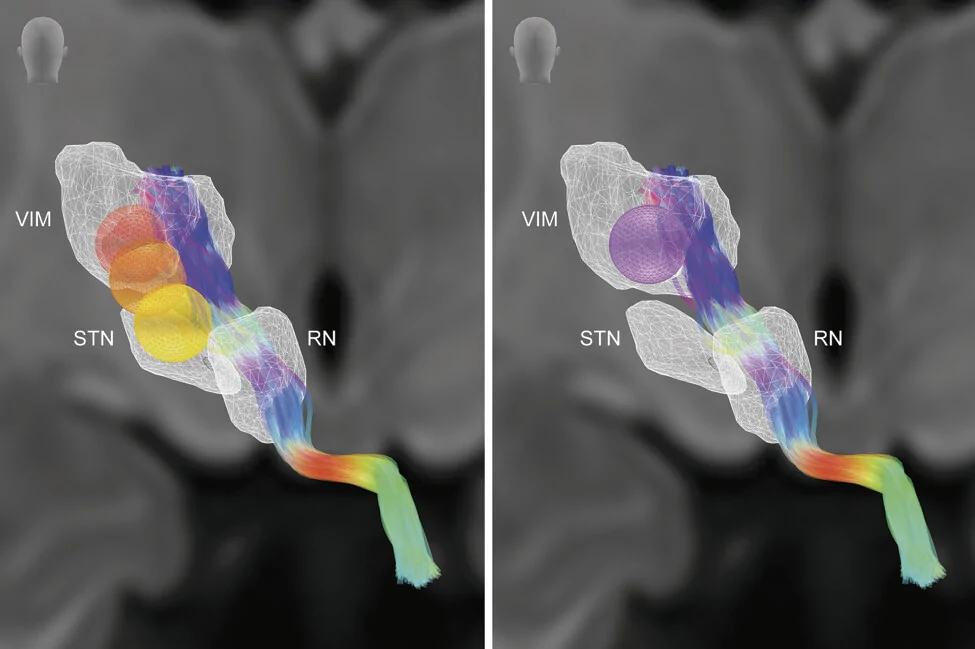
Averaged stereotactic targets for tremor therapy (deep brain stimulation [DBS] vs. MR-guided focused ultrasound [MRgFUS]). Commonly used DBS stimulation sites (left) for tremor control within the classical ventral intermediate nucleus (VIM) target (red circle). Posterior subthalamic area (PSA, yellow) and zona incerta (ZI, orange) are the two white matter targets within a stronger tremor, located close to the dentatorubrothalamic tract (DRTT, rainbow-colored streamlines). The MRgFUS (right) thalamotomy lesion centers inside the VIM (purple circle). RN, red nucleus; STN, subthalamic nucleus.

The therapeutic “sweet spot” of MRgFUS is generally located in the VIM of the thalamus, slightly posterior and lateral to the midpoint of the thalamus, where lesioning most effectively disrupts tremor activity 34 ( Fig. 10 ). This differs in emphasis from DBS, where the most beneficial stimulation sites often extend beyond the VIM into the PSA and ZI, regions rich in fiber tracts such as the DRTT. In other words, it seems like MRgFUS is constrained to lesioning a focal thalamic nucleus, whereas DBS can modulate distributed white matter pathways within the tremor network. This mechanistic difference helps explain why MRgFUS appears more nucleus-focused, while DBS has evolved toward tract-based targeting—two complementary strategies for engaging the same cerebello-thalamo-cortical tremor circuit.

## Conclusion

Neuroimaging underpins both diagnosis and treatment of tremor syndromes. In routine workup, MRI is the modality of choice to exclude structural causes, while molecular imaging—especially presynaptic dopaminergic SPECT/PET—helps distinguish neurodegenerative parkinsonism from non-dopaminergic deficiency tremor (e.g., ET), but is not useful for differentiating between parkinsonian subtypes. For surgery, imaging spans the entire pathway: preoperative MRI for target planning and risk assessment; intraoperative fluoroscopy/CT/iMRI for lead or lesion guidance and safety; and postoperative CT/MRI fused to preoperative scans for verification, programming, and outcome analysis. Group analyses of such current data define optimal targets for both DBS and MRgFUS, providing refined guidance for targeting and programming based on population-level imaging and connectivity data. Overall, a pragmatic algorithm integrates structural MRI for all atypical or combined tremors, dopaminergic imaging when parkinsonism is suspected, and advanced imaging to guide and optimize neurosurgical therapies.

## Declaration of GenAI Use

During the preparation of this work the authors used ChatGPT-4o (OpenAI, USA) in order to assist with language-related tasks. After using this tool/service, the authors reviewed and edited the content as needed and take full responsibility for the content of the publication.
